# Supplementary material for: Low-rank Gallus gallus domesticus chicks are better at transitive inference reasoning
Source: Commun Biol. 2021 Dec 9;4:1344. doi: 10.1038/s42003-021-02855-y (PMC8660828; doi:10.1038/s42003-021-02855-y)
Supplement: Supplementary file 2 — Reporting Summary [file 42003_2021_2855_MOESM2_ESM.pdf]

nature research

Corresponding author(s): Lucia Regolin

Last updated by author(s): Sep 12, 2021

Reporting Summary

Nature Research wishes to improve the reproducibility of the work that we publish. This form provides structure for consistency and transparency in reporting. For further information on Nature Research policies, see our Editorial Policies and the Editorial Policy Checklist.

Statistics

For all statistical analyses, confirm that the following items are present in the figure legend, table legend, main text, or Methods section.

n/a	Confirmed	
☐	☒	The exact sample size (*n*) for each experimental group/condition, given as a discrete number and unit of measurement
☒	☐	A statement on whether measurements were taken from distinct samples or whether the same sample was measured repeatedly
☐	☒	The statistical test(s) used AND whether they are one- or two-sided<br>*Only common tests should be described solely by name; describe more complex techniques in the Methods section.*
☒	☐	A description of all covariates tested
☐	☒	A description of any assumptions or corrections, such as tests of normality and adjustment for multiple comparisons
☐	☒	A full description of the statistical parameters including central tendency (e.g. means) or other basic estimates (e.g. regression coefficient) AND variation (e.g. standard deviation) or associated estimates of uncertainty (e.g. confidence intervals)
☐	☒	For null hypothesis testing, the test statistic (e.g. *F*, *t*, *r*) with confidence intervals, effect sizes, degrees of freedom and *P* value noted<br>*Give P values as exact values whenever suitable.*
☒	☐	For Bayesian analysis, information on the choice of priors and Markov chain Monte Carlo settings
☒	☐	For hierarchical and complex designs, identification of the appropriate level for tests and full reporting of outcomes
☒	☐	Estimates of effect sizes (e.g. Cohen's *d*, Pearson's *r*), indicating how they were calculated

Our web collection on statistics for biologists contains articles on many of the points above.

Software and code

Policy information about availability of computer code

Data collection	Data collection was carried out manually - i.e. no digital capture
Data analysis	SPSS 13.0 for windows

For manuscripts utilizing custom algorithms or software that are central to the research but not yet described in published literature, software must be made available to editors and reviewers. We strongly encourage code deposition in a community repository (e.g. GitHub). See the Nature Research guidelines for submitting code & software for further information.

Data

Policy information about availability of data

All manuscripts must include a data availability statement. This statement should provide the following information, where applicable:

- Accession codes, unique identifiers, or web links for publicly available datasets
- A list of figures that have associated raw data
- A description of any restrictions on data availability

Data uploaded to figshare with DOI 10.6084/m9.figshare.13027829

Figure 1b Choice of B in the comparison BD with respect to rank and to sex,
with raw data in provided Excel sheet

No restrictions on data availability

Field-specific reporting

Please select the one below that is the best fit for your research. If you are not sure, read the appropriate sections before making your selection.

☒ Life sciences ☐ Behavioural & social sciences ☐ Ecological, evolutionary & environmental sciences

For a reference copy of the document with all sections, see nature.com/documents/nr-reporting-summary-flat.pdf

Life sciences study design

All studies must disclose on these points even when the disclosure is negative.

Sample size	No pre-determination of sample size was carried out. Sample size was constrained by the limitations of space (within the laboratory) and time (the training/testing procedure and numbers of chicks associated with the process to allow student projects to progress). The sample size obtained was considered the minimum (ostensibly an n=6 for ranked female birds) to allow parametric statistics of normalised values (ln) to be carried out. Previous studies using similar methodology also employed similar samples. Keeping the sample to the minimum possible number of subjects complies with the principle of Reduction in animal research.
Data exclusions	No data were excluded from analyses.
Replication	No attempt has been made to reproduce the experiment per se, however previous work in the laboratories at Padova has shown that chicks are able to perform transitive inference and that lateralization of the task is present in male chicks (Daisley et al., 2010).
Randomization	Chicks were raised individually for the first 4 days of life. On the 5th day same-sex chicks were taken, by random choice, to a larger cage into which 3 (or sometimes 4) individuals were placed together. From this point the chicks remained as a group throughout the experiment.
Blinding	The experiment was not blind given that the experimenters (JD together with a master's student) were involved in both intensive training and testing the, relatively small number of, chicks throughout. No expectations regarding the direction of the null hypothesis (rank affects TI) was anticipated, however, with the outcome of lower ranked chicks performing better than higher ranking ones unexpected.

Reporting for specific materials, systems and methods

We require information from authors about some types of materials, experimental systems and methods used in many studies. Here, indicate whether each material, system or method listed is relevant to your study. If you are not sure if a list item applies to your research, read the appropriate section before selecting a response.

Materials & experimental systems

n/a	Involved in the study	
☒	☐	Antibodies
☒	☐	Eukaryotic cell lines
☒	☐	Palaeontology and archaeology
☐	☒	Animals and other organisms
☒	☐	Human research participants
☒	☐	Clinical data
☒	☐	Dual use research of concern

Methods

n/a	Involved in the study	
☒	☐	ChIP-seq
☒	☐	Flow cytometry
☒	☐	MRI-based neuroimaging

Animals and other organisms

Policy information about studies involving animals; ARRIVE guidelines recommended for reporting animal research

Laboratory animals	Gallus gallus domesticus: 44 Hybro chicks (26 males), aged: from hatching to Day 20 post hatching.
Wild animals	The study did not involve wild animals.
Field-collected samples	The study did not involve samples collected from the field.
Ethics oversight	All of the experiments adhered to the Italian and European Union directives on animal research, University of Padova License: CEASA prot. 37/2011, Ministry of Health License: 6/2012-B (10-01-2012).

Note that full information on the approval of the study protocol must also be provided in the manuscript.
